# Fungicide Drives *De Novo* Evolution of Multidrug Resistance in the Plant Growth Promoting Rhizobacterium, *Pseudomonas fluorescens*

**DOI:** 10.1111/eva.70307

**Published:** 2026-08-02

**Authors:** Matthew Kelbrick, James P. J. Hall, Siobhán O'Brien

**Affiliations:** ^1^ Department of Evolution, Ecology and Behaviour, Institute of Infection, Veterinary and Ecological Sciences University of Liverpool Liverpool UK; ^2^ Moyne Institute of Preventive Medicine, Department of Microbiology, School of Genetics and Microbiology Trinity College Dublin Dublin 2 Ireland

**Keywords:** adaptation, antibiotic resistance, bacteria, evolution, experimental evolution, fungicide

## Abstract

Soil microbial communities underpin key ecosystem processes, including plant health, nutrient cycling and primary productivity. In agricultural soils, beneficial soil bacteria are often exposed to multiple stressors simultaneously, including fungicides, antibiotics, and warming temperatures. Despite their ecological importance, little is known about how beneficial soil bacteria respond to such combined stressors over evolutionary timescales. Here, we investigated how the model plant growth promoting rhizobacterium (PGPR) *Pseudomonas fluorescens* evolves resistance to the fungicide formulation Fubol Gold (metalaxyl‐M + mancozeb), under ambient or warming soil conditions. Using a 16‐week evolution experiment in soil microcosms with a fully factorial design (fungicide ± warming), we assessed the evolution of fungicide resistance via phenotypic assays and whole‐genome sequencing. Fungicide exposure rapidly selected for increased fungicide resistance, detectable as early as week 4, and co‐selected for resistance to chloramphenicol, sulphatriad and nalidixic acid antibiotics, likely through mutations in a *mexS* ortholog that may be associated with efflux pump overexpression. Warming did not alter the evolution of fungicide resistance; however, populations subjected to both fungicide and warming stress went extinct more rapidly, so that population evolutionary rescue was less effective under dual stress. Our findings show that fungicide alone can drive multidrug resistance in beneficial soil bacteria, suggesting that non‐antibiotic drivers of resistance in the environment should be incorporated into One Health frameworks for tackling AMR.

## 1 Introduction

Fungicides, including azoles, dithiocarbamates and phenylamides, are widely applied in agricultural systems to control plant‐pathogenic fungi and safeguard crop yields (Brauer et al. 2019; Fenner et al. 2013). Although designed to target fungal pathogens, fungicides inevitably enter soil environments where they interact with diverse non‐target microorganisms (Fenner et al. 2013). Soil bacterial communities play essential roles in nutrient cycling, organic matter turnover and plant health (Bardgett and van der Putten 2014), and there is increasing evidence that fungicide application can alter soil community structure and function. Experimental and field studies show that fungicides shift bacterial community composition, reduce alpha diversity and disrupt microbial interaction networks in agricultural soils (Kelbrick et al. 2026; Sliti et al. 2024; Tagele and Gachomo 2025). Plant growth‐promoting rhizobacteria (PGPR) are a functionally important group of soil bacteria that enhance plant productivity through nutrient mobilisation, phytohormone production and pathogen suppression (Wahab et al. 2025). Fungicide resistance has been identified in several PGPR taxa, raising the possibility that resistant strains could buffer crops against the negative side effects of chemical disease control (Al‐Enazi et al. 2022; Khan et al. 2020). However, the ecological and evolutionary dynamics underpinning fungicide resistance in PGPR, especially under future climate conditions, remain poorly understood.

Agricultural soils are inherently multistressor environments in which microorganisms are exposed not only to pesticides but also to fertiliser inputs, heavy metals and rising temperatures associated with climate change (Kelbrick et al. 2023). Adaptation under multiple stressors can be constrained by trade‐offs, such as antagonistic pleiotropy, where mutations that confer resistance to one stressor reduce tolerance to another (Anderson et al. 2013). Genetic correlations, epistasis, and population bottlenecks may further limit adaptive potential (Ostman et al. 2012). Conversely, resistance to multiple stressors may arise through shared physiological mechanisms, such as the upregulation of multidrug efflux pumps (Amsalu et al. 2020; Poole 2012; Wand et al. 2022) or broad‐spectrum hydrolases capable of degrading diverse xenobiotics (Kirubakaran et al. 2019). Co‐regulation among stress response pathways may also accelerate adaptation when exposure to one stressor primes tolerance to another, as has been observed for elevated temperature and certain antibiotic classes (Rodriguez‐Verdugo et al. 2020). Despite these contrasting possibilities, we lack empirical tests of how warming temperatures shape fungicide resistance evolution in beneficial soil bacteria.

Here, we investigate whether warming temperatures constrain fungicide resistance evolution in the model PGPR *Pseudomonas fluorescens* using experimental evolution in compost soil microcosms (Hall et al. 2017; Hall et al. 2018). Our microcosm system mimics the spatial structure and resource heterogeneity of natural soils while maintaining high replication and experimental control under laboratory conditions. We first identify a fungicide formulation that has a strong inhibitory effect on *P. fluorescens* (Fubol Gold‐ a fungicide formulation containing the phenylamide metalaxyl‐M and the dithiocarbamate mancozeb, applied to control oomycete pathogens such as potato late blight). Next, we use experimental evolution to test whether warming temperatures constrain fungicide resistance, using a combination of phenotypic assays and whole genome resequencing on evolved and ancestral PGPR. Finally, we test whether fungicide‐evolved isolates display cross‐resistance to a panel of antibiotics. Our findings show that the fungicide Fubol Gold drives selection for both fungicide and antibiotic resistance in *P. fluorescens*, irrespective of warming.

## 2 Methods

### 2.1 Bacterial Strains

For experimental evolution, we used *P. fluorescens* SBW25 carrying a gentamicin‐resistance cassette (SBW25::Gm^R^) (Hall et al. 2015). Because the ancestral strain showed poor survival in soil, we instead used a SBW25::Gm^R^ isolate that had previously adapted to John Innes No. 2 soil‐based compost. This isolate, A.21.65. G.003, was retrieved from a plasmid‐free population of *P. fluorescens* SBW25 that had evolved alongside *P. putida* KT2440 in control soil microcosms as described in (Hall et al. 2017; Hall et al. 2018). Previous whole‐genome sequencing (Short Read Archive accession number ERR1554495) identified three nonsynonymous mutations in this soil‐adapted SBW25::Gm^R^ relative to the reference SBW25 genome: Phe133Val in PFLU_5421 (putative peptidoglycan‐related gene), Asp225Ala in PFLU_6004 (conserved hypothetical), and Thr128Pro in PFLU_0941 (putative peptidoglycan glycosyltransferase) (Hall et al. 2018), which were also detected in all eight ancestral clones in our evolution experiment (Supporting Information S1). In preliminary assays, the soil‐adapted SBW25::Gm^R^ was recoverable in all replicates (*n* = 12).

### 2.2 Quantifying Inhibitory Effects of Four Different Fungicide Formulations on Bacterial Growth

We assessed the effects of four fungicides available for agricultural use in EU/UK when this work was carried out: *Amistar* (Syngenta; azoxystrobin), *Fubol Gold* (Syngenta; mancozeb, metalaxyl‐M), *ProPlant* (Dejex; propamocarb hydrochloride), and *Subdue* (Dejex; metalaxyl‐M) on *P. fluorescens* final densities in soil microcosms. A quantity of 10 g of John Innes No. 2 soil (J. Arthur Bowers) was placed in 30 mL glass vials, autoclaved twice at 126°C for 15 min, and left at room temperature for 5 days to allow reoxidation of heat‐generated toxic compounds (Sonneveld and Voogt 1975). Fungicides were mixed with sterile distilled water at a 100:1 (w/w) ratio. Each soil microcosm received 900 μL fungicide solution, giving a final concentration of 0.1% (w/w) in soil. Control microcosms received 900 μL sterile water. Three replicate microcosms were prepared per treatment (15 microcosms total), vortexed for 30 s, and equilibrated for 1 h.

A frozen stock of *P. fluorescens* was streaked onto Lysogeny Broth Agar (LBA) and incubated at 26°C for 48 h. A single colony was cultured in 10 mL Lysogeny Broth (LB) at 26°C, 180 rpm for 24 h, centrifuged (3000 × g, 10 min), washed twice in M9 buffer, and resuspended in 10 mL M9. Each soil microcosm was inoculated with 100 μL of this culture (10^5^ colony‐forming units (CFUs) mL^−1^) so the starting density of *P. fluorescens* was 10^3^ CFUs g^−1^ soil. Microcosms were vortexed for 30 s and incubated at 26°C and 70% humidity for 4 days. After incubation, 20 sterile glass beads (5 mm; Witeg) and 10 mL M9 buffer were added, vortexed for 45 s, and left for 30 min to facilitate the separation of the supernatant from the soil wash. The supernatant was serially diluted and plated (50 μL) on LBA containing 25 μg mL^−1^ gentamicin (Melford) to ensure selective growth of our *P. fluorescens* strain. Plates were incubated for 48 h at 26°C, and CFUs g^−1^ soil were enumerated. All fungicide formulations significantly decreased *P. fluorescens* final densities compared to fungicide‐free controls (Fubol Gold; *t*‐test: t = 143.73, *p.ad*j < 0.001; Amistar; *t*‐test: *t* = 28.955, *p.adj* < 0.001, Subdue; *t* = 33.379, *p.adj* < 0.001, ProPlant; *t*‐test: *t* = 20.645, *p.adj* < 0.001, Figure S1). For our remaining experiment, we chose to focus on Fubol Gold, as it had the strongest inhibitory effect on *P. fluorescens*.

### 2.3 Experimental Evolution of *P. fluorescens* in Soil

*P. fluorescens* was experimentally evolved in sterile soil microcosms under four treatments: control, fungicide, warming, and fungicide + warming, using a fully factorial design (8 replicates per treatment, 32 microcosms in total). Fungicide concentrations were increased every 4 weeks (week 1–4: 10 mg kg −1 (0.001%)); week 4–8: 50 mg kg −1 (0.005%); week 8–12: 100 mg kg −1 (0.01%); week 12–16: 500 mg kg −1 (0.05%) (w/w). Preliminary experiments showed a ~6000‐fold reduction in *P. fluorescens* densities at 0.1% Fubol Gold; therefore, concentrations were kept below this level during the evolution experiment to prevent extinction. Warming‐treated microcosms increased from 26°C in increments of 0.5°C every 7 days, until a maximum temperature of 34°C at week 16.

A 1 g mL^−1^ Fubol Gold stock was prepared in sterile distilled water and diluted to the desired concentration, starting at 10 mg kg^−1^ soil (0.001% w/w) at week 1. Sixteen 10 g sterile soil microcosms received 900 μL of fungicide solution, and controls received sterile water. Microcosms were vortexed for 30 s and equilibrated for 1 h before inoculation.

A frozen stock of *P. fluorescens* was streaked on LBA supplemented with 25 μg mL^−1^ gentamicin and incubated for 48 h at 26°C. Eight colonies were randomly selected and cultured individually in 10 mL LB at 26°C with shaking (180 rpm) for 24 h, centrifuged (3000 × g, 10 min), washed twice with M9 buffer, and resuspended in 10 mL M9. A volume of 100 μL (10^5^ CFU mL^−1^) of each clone was inoculated into four soil microcosms each (one soil microcosm per treatment) so final bacterial density was 10^3^ g^−1^ soil. Microcosms were vortexed for 30 s and incubated at 26°C and 70% relative humidity.

Populations were passaged every 7 days for 16 weeks (112 days). A volume of 10 mL M9 buffer and 20 sterile glass beads were added to each microcosm, vortexed for 45 s, and allowed to settle for 30 min. A 100 μL aliquot of the soil wash was transferred to fresh microcosms with the appropriate fungicide concentration and vortexed for 30 s. Every 4 weeks, 150 μL of soil wash was cryopreserved in 20% (w/v) glycerol at −80°C. Population densities (CFU g^−1^ soil) were quantified every 4 weeks by serial dilution of 20 μL soil wash in M9, plating on gentamicin‐supplemented LBA, and incubating for 24 h. To verify identity of our inoculated SBW25::Gm^R^ strain, 12 clones (3 per treatment) were selected at random and confirmed as SBW25::Gm^R^ by PCR targeting the mini‐Tn7 GentR cassette (Supporting Information).

### 2.4 Genome Resequencing Evolved and Ancestral *P. fluorescens* Clones

To identify treatment‐specific mutational patterns, 2 colonies were selected at random from each population at the final time point (week 16); for populations that went extinct earlier, clones were sampled from the last viable time point. All eight ancestral clones were also sequenced. Frozen population stocks were thawed, serially diluted, plated on LBA, and incubated at 26°C for 48 h. Two colonies per plate were cultured in 5 mL LB (26°C, 180 rpm, 24 h) and cryopreserved (1 mL culture mixed with 500 μL of 60% w/v glycerol, −80°C). Clones were re‐streaked on LBA, incubated for 48 h, and biomass was harvested into 500 μL DNA/RNA Shield (MicrobesNG). Genomic DNA was extracted following MicrobesNG protocols and sequenced on an Illumina NovaSeq 6000 platform (2 × 250 bp; > 30× coverage). Reads were trimmed with Trimmomatic v0.30 and quality‐checked using FastQC. Variants were called with breseq v0.37.1 (Deatherage and Barrick 2014) against the *P. fluorescens* SBW25 reference genome (AM181176). Two contaminant (non‐Pseudomonas) clones (both from warming population 4) were excluded from analysis. All reads have been uploaded to NCBI (Accession: PRJNA1471490, BioProject ID: 1471490).

### 2.5 Quantifying Fungicide Minimum Inhibitory Concentrations for Evolved and Ancestral Clones

To test whether (i) clones from fungicide‐evolved treatments differed in fungicide resistance versus control‐evolved clones and (ii) whether fungicide‐evolved clones carrying mutations in *PFLU_3160* exhibited increased fungicide resistance compared to wildtype, we performed a minimum fungicide inhibitory concentration (MIC) assay. One clone was selected at random from each fungicide‐evolved, fungicide+warming evolved, control‐evolved and ancestral populations (32 clones in total, 8 clones per treatment). These clones also varied with respect to *PFLU_3160* mutations (13/16 clones had *PFLU_3160* mutation in fungicide treatments, while all ancestral and control clones had wildtype *PFLU_3160*). Clones were streaked from frozen stocks onto LBA plates and incubated for 48 h at 26°C. Several colonies from each plate were collected using sterile cotton swabs and suspended in 10 mL 0.85% (w/v) saline to achieve a cell density equivalent to a McFarland 0.5 standard.

A 65.5 mg mL^−1^ solution of fungicide was prepared in ×2 LB broth (diluted to ×1 when culture in saline was added). The fungicide solution was serially diluted in a 96‐well plate, with each dilution transferring 100 μL fungicide solution into 100 μL LB (for a 2‐fold dilution series); this was repeated 24 times until fungicide concentration was reduced to 0.015625 μg mL^−1^. A 100 μL sample was removed from the final well and discarded. This dilution series was replicated four times, three as experimental replicates and one as an uninoculated control. A 100 μL sample of standardised bacterial culture in 0.85% saline (or 0.85% saline for controls) was added to each of the wells (diluting LB to ×1). Thus, the final fungicide concentration in wells was between 32.25 mg mL^−1^ and 0.0078 μg mL^−1^ in two‐fold increments. Plates were incubated at 26°C and 600RPM for 24 h using a microplate shaker (Stuart) at 600 RPM. Growth inhibition was visually assessed, with the lowest fungicide concentration showing no visible growth (clear well) recorded as the MIC in μg mL^−1^ for each clone.

### 2.6 Quantifying Antibiotic Resistance for Evolved and Ancestral Clones

We tested whether (i) fungicide evolved clones had increased antimicrobial resistance (AMR) compared to control‐evolved clones, and (ii) whether *PFLU_3160* mutants had increased resistance compared to wildtype, using disk diffusion assays in accordance with EUCAST guidelines (Matuschek et al. 2014). Clones were standardised to a cell density equivalent to a McFarland 0.5 standard and, using a sterile cotton swab, cultures were spread onto square agar plates (50 mL LBA) in three directions to ensure complete coverage. Then, either MAST antibiotic rings (impregnated with Colistin Sulphate 25 μg, Streptomycin 10 μg, Sulphatriad 200 μg or Tetracycline 25 μg) or disks (impregnated with Nalidixic acid 30 μg or Chloramphenicol 30 μg) were firmly placed onto the agar surface using autoclaved sterile tweezers. Each disk assay was repeated three times per clone. Plates were incubated for 24 h at 26°C. After 24 h, inhibition halos were measured to the nearest millimetre using a ruler.

### 2.7 Statistical Analysis

- *Fungicide inhibition tests*: To assess the effects of four different fungicide formulations on *P. fluorescens* growth, we conducted pairwise *t*‐tests on log_10_‐transformed CFU g^−1^ soil data, comparing each formulation with the fungicide‐free control. *p*‐values were adjusted for multiple comparisons using Bonferroni correction.
- *Population densities over time*: Population extinction dynamics were analysed using Kaplan–Meier survival curves, with extinction treated as the event and surviving populations right‐censored at the final sampling point. Extinction was defined as the absence of viable cells by plating. Kaplan–Meier survival curves were estimated using the survfit() function in the survival package (Therneau 2023) and visualised using ggsurvplot() in the survminer package (Kassambara and Biecek 2025). Differences in survival among warming and fungicide treatments were assessed using log‐rank tests (survdiff() function in the survival package), which account for both the frequency and timing of extinction events. The pairwise_survdiff() function from the survminer package was used to test for pairwise comparisons between treatments, with Bonferroni correction for multiple testing.
- *Genome resequencing*: To accommodate mutations in our soil‐adapted SBW25 ancestor relative to the reference genome, and to control for spurious prediction of mutations in repetitive or difficult‐to‐map parts of the reference, any mutations identified in our 8 ancestral clones relative to the SBW25 reference sequence, including mutations with ‘marginal’ or ‘unassigned’ evidence, were pooled and removed from all clones in the evolved variant dataset prior to statistical analysis. To detect if mutational patterns among evolved clones were treatment specific, we used a factorial PERMANOVA with 10,000 permutations using the adonis() function from the ‘vegan’ v 2.6–4 package in R (Oksanen et al. 2025). Significance of individual model terms was assessed using marginal tests (by = “terms”). Homogeneity of multivariate dispersion among treatment groups was evaluated using the betadisper() function, followed by ANOVA, to confirm that significant PERMANOVA results were not driven by differences in within‐group variance.

To identify putative treatment‐specific parallel mutations, we identified candidate mutations as those which appeared in > 1 population within a treatment and 0 in the alternative treatment. Directly adjacent genes belonging to the same operon with overlapping functions (e.g., *actP* and *PFLU_1813*) were grouped and treated as a single mutational target. Candidates were tested using Fisher's exact test, with *p*‐values corrected for multiple testing using a Bonferroni‐adjustment. In our dataset of 7 parallel targets, two targets consisted of large deletions in intergenic regions including (i) a putative 133 bp deletion in the intergenic region *PFLU_2441‐PFLU_2442* (downstream of two LysR family transcriptional regulators), detected in 32/62 clones across all 4 treatments, and (ii) a putative 167–249 bp deletion in intergenic region *PFLU_4254‐ PFLU_4255* (a transposase and an integral membrane protein coding gene respectively; 11/62 clones across all 4 treatments). Notably, these deletions were predicted in repeat‐rich regions prone to spurious mutation calls (Deatherage and Barrick 2014; Hall et al. 2020) so were removed from our final dataset of parallel mutational targets.

iv *Resistance assays*. Mode fungicide MIC (3 replicates per clone) was taken as the MIC for each clone. We tested for differences in log_2_ fungicide MIC between evolution treatments (control, fungicide and fungicide+warming) using ANOVA and Tukey tests, confirming normality of residuals using QQ‐plots and Shapiro–Wilk normality tests (*W* = 0.95). Differences in log_2_ MIC between *PFLU_3160* mutants and wildtype clones was tested using a Welch two sample *t*‐test, to allow for unequal variances between treatments. To test the relative contributions of evolutionary treatment and mutation status on fungicide MIC, we used a linear model (log_2_ MIC ~ treatment + mutation status) and likelihood ratio tests.

We tested whether antimicrobial resistance (quantified as the mean zone of inhibition in millimetres) differed between treatments (control, fungicide, and fungicide + warming) using Kruskal‐Wallis with Bonferroni correction and Dunn posthoc tests. All clones (including ancestral strains) were completely resistant to ampicillin, cephalothin, and cotrimoxazole (zone of inhibition = 0 mm for all clones); these antibiotics were therefore excluded from statistical analyses. Differences in resistance between *PFLU_3160* mutants and wildtype clones were tested for each antibiotic using Wilcoxon Rank‐Sum tests with Bonferroni correction.

## 3 Results

### 3.1 Dual Stressors Accelerate Population Extinction

Treatment (fungicide, warming, dual stressors or stress‐free control) significantly affected extinction risk in our evolving populations (log‐rank test: χ^2^₃ = 31.5, *p* < 0.0001, Figure S2). Populations in the control treatment remained fully viable throughout the experiment, with densities consistently high, ranging from 3.0 × 10^8^ to 9.0 × 10^9^ CFU g^−1^ soil (Figure S3). All single‐stressor populations remained viable until week 16, when extinction occurred in all fungicide populations and 4/8 warming populations. In contrast, dual‐stressor populations had a median survival of 12 weeks, with 2/8 populations reaching extinction by week 8. When stressors were imposed independently, fungicide increased extinction risk relative to controls (*p* < 0.001), but warming did not (*p* > 0.05). Dual stressor treatments had increased extinction risk relative to controls (*p* < 0.001), fungicide only (*p* < 0.05) and warming only (*p* < 0.01) (Figure S2). Our data shows that extinction rates in dual‐stressor populations could not have been predicted from single‐stressor dynamics alone.

### 3.2 Pinpointing Genetic Responses to Selection in a Multi‐Stressor Environment

We hypothesised that, prior to extinction, populations could evolve by acquiring mutations to adapt to the increasing stress. To identify treatment‐specific patterns of mutation, we selected two clones from each evolved population at the final time point prior to extinction for whole‐genome resequencing. Across evolved clones, we detected 152 mutations absent in ancestral clones, comprising gene deletions (53%), insertions (11%) and single nucleotide polymorphisms (37%), including non‐synonymous (45%), synonymous (16%), intergenic (9%) and nonsense (30%) (Supporting Information S1, Figure S4). Evolved clones had between 0 and 6 mutations with a median of 2 mutations per clone. We did not detect any mutations in one clone (B8Y; fungicide‐only treatment).

**FIGURE 1:**
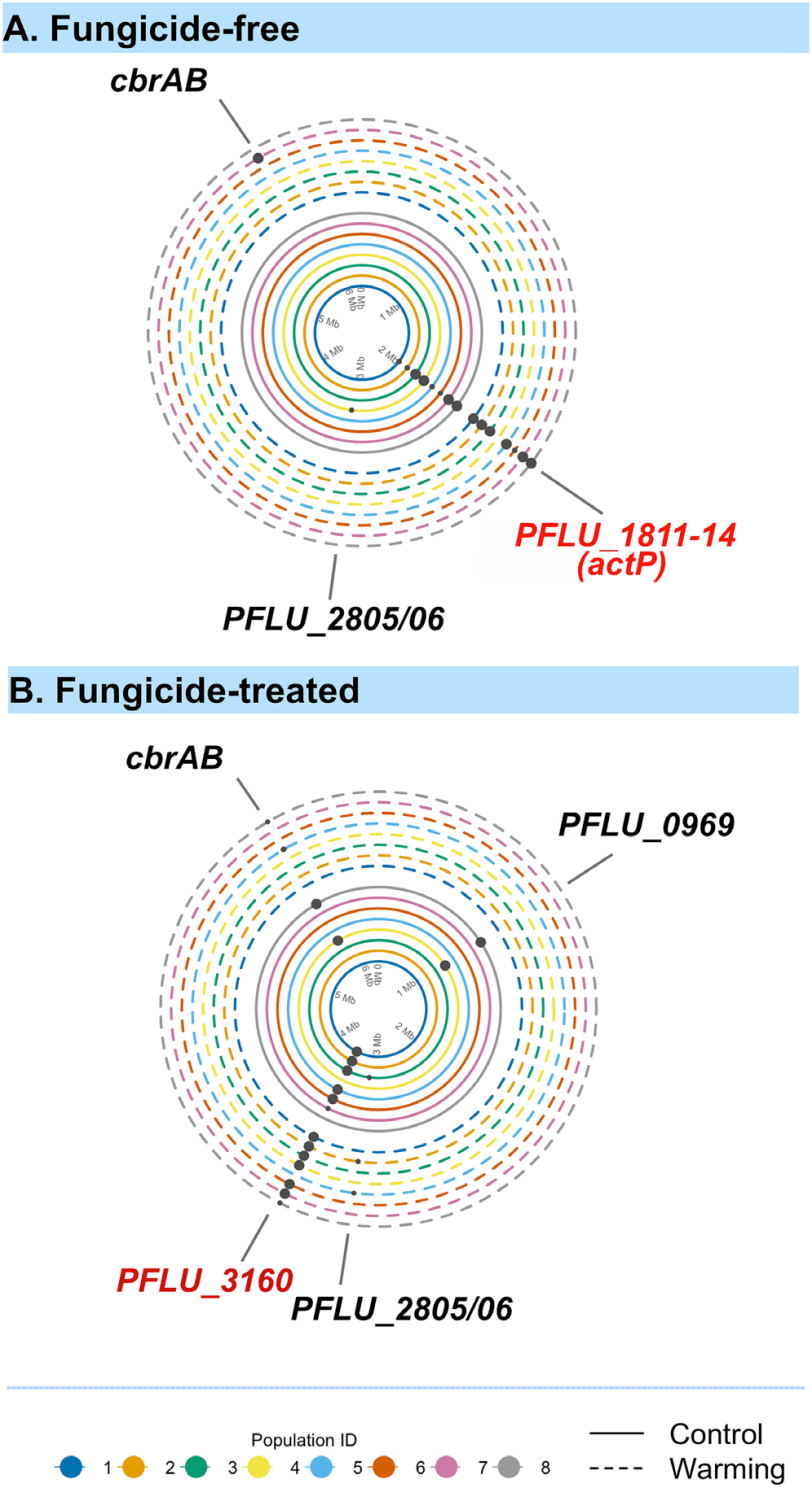
Mutations under positive selection as indicated by parallel evolution in *P. fluorescens* populations evolved in fungicide‐free (A) or (B) fungicide‐treated soil. Each concentric circle corresponds to a replicate population in either the control (without fungicide) or treatment group (with fungicide). Warmed and unwarmed populations are shown as broken lines and solid lines, respectively. Positions around each concentric circle, starting at the 12 o'clock position and in a clockwise direction, correspond to positions around the published *P. fluorescens* SBW25 single circular chromosome. Black points show the position of mutations; only those mutated in parallel (i.e., in > 1 replicate population) are shown here. Point size corresponds to the number of clones harbouring the specific mutation per population (i.e., one or two sequenced clones). Mutation labels coloured red are significantly associated with fungicide treatment: (*actP*, unique to fungicide‐free populations) and *PFLU_3160* (unique to fungicide‐treated populations). Mutations in *PFLU_1813*, *PFLU_1814 (actP)* and a large deletion in*PFLU_1811‐actP* are grouped for clarity. A full list of mutations is available in supporting information (S1 and S2).

We identified mutations in 36 distinct loci across all evolved clones, with 7 targets mutated in more than one population (i.e., parallel evolution) (Figure 1, Supporting Information S2). Genome‐wide patterns of mutation were significantly associated with fungicide treatment (permutational MANOVA: effect of fungicide, R^2^ = 0.26, F_1,27_ = 10.1, *p* < 0.0001), irrespective of warming (fungicide × warming, R^2^ = 0.02, F_1,27_ = 0.9, *p* > 0.05). We could not detect any significant statistical association between warming and the pattern of mutations (effect of warming, R^2^ = 0.02, F_1,27_ = 0.8, *p* > 0.05).

Treatment‐specific parallel evolution can suggest natural selection acting upon a specific gene or genes (Scheuerl et al. 2020). From our dataset of 5 parallel targets, two targets were significantly associated with fungicide treatment: (i) *PFLU_3160*, unique to fungicide‐treated populations and (ii) *actP—*occurring only in non‐fungicide treatments (Fisher's exact test, Bonferroni‐adjusted: *p* < 0.0001 for both targets, Figure 1). Mutations in *PFLU_3160* were identified in 24/32 clones across 13/16 fungicide‐evolved populations and were detected as early as week 4 (fungicide+warming populations 1 + 5). *PFLU_3160* is a *P. aeruginosa* *mexS* ortholog which can regulate the expression of efflux pumps and porins (Morita et al. 2015; Uwate et al. 2013). In contrast, mutations in *actP* or adjacent *PFLU_1813* were unique to fungicide‐free populations, appearing in 23/30 clones across 15/16 populations. Mutations in *actP* have been previously shown to be advantageous in this soil environment (Hall et al. 2018), yet our findings suggest *actP* mutations are constrained in the presence of fungicide. Warming had no effect on the distribution of each of the 5 parallel targets across treatments (Fisher's exact test, *p* > 0.05 in all cases, Figure 1).

### 3.3 Selection for Increased Fungicide‐Resistance

**FIGURE 2:**
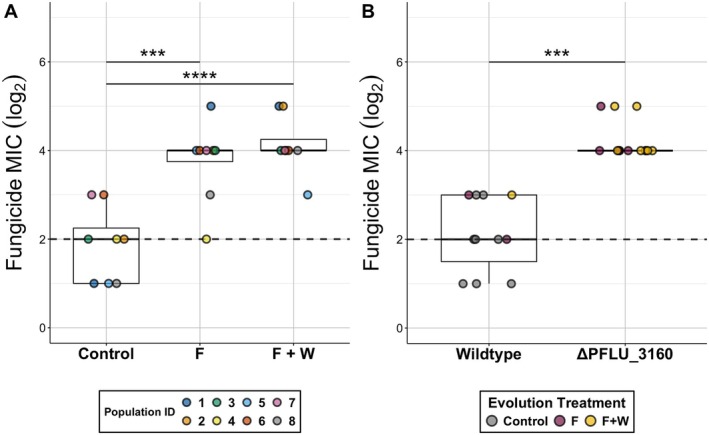
Fungicide resistance in evolved isolates. (A) Log_2_ fungicide MIC for isolated *P. fluorescens* clones evolved under control, fungicide (F) or fungicide+warming (FT) treatments. Points are coloured according to population ID. (B) The same dataset grouped by *PFLU_3160* mutation (ancestral or mutated). Points are coloured by evolutionary treatment. Individual points represent mode MIC for 3 technical replicates per clone. Dashed red horizontal line shows mode Log_2_MIC value for the ancestor. Boxplots show the median (central line), interquartile range (box), and 1.5× interquartile range (whiskers). Lines connecting groups indicate significant differences between groups. Significance levels are denoted as: *P* < 0.05 (*), *p* < 0.01 (**), *p* < 0.001 (***), *p* < 0.0001 (****).

Fungicide MIC was significantly affected by evolution treatment (ANOVA: F = 18.43, *p* < 0.0001, Figure 2a). Fungicide‐evolved clones had increased fungicide MICs compared to fungicide‐free controls, irrespective of whether fungicide was applied alone or alongside warming (TukeyHSD; fungicide versus control: *p* < 0.001; fungicide + warming versus control: *p* < 0.0001, Figure 2a). Fungicide adaptation was not constrained by warming; there was no difference in MIC between fungicide populations evolved under control versus warming conditions (TukeyHSD; fungicide versus fungicide+warming: *p* > 0.5). For all tested clones, MICs ranged from 2 to 32 μg mL^−1^. Ancestral and control‐evolved clones had an MIC of 2–8 μg mL^−1^, while fungicide‐evolved clones had an MIC ranging from 4 to 32 μg mL^−1^ (Table S1).

Our sequencing results suggested that mutations in *PFLU_3160* may be associated with increased resistance to fungicide. We compared fungicide MIC specifically for clones harbouring a *PFLU_3160* mutation versus clones with wildtype *PFLU_3160* (Figure 2b, Table S1). Our dataset comprised fungicide‐evolved clones with *PFLU_3160* mutation (*n* = 13), (ii) fungicide‐evolved clones with wildtype *PFLU_3160* (*n* = 3), (iii) control (no‐fungicide) evolved clones with wildtype *PFLU_3160* (*n* = 8), and (iv) ancestral clones of each population with wildtype *PFLU_3160* (*n* = 8). *PFLU_3160* mutants had significantly increased MIC compared to wildtype overall (*t*‐test, *t* = 7.68, *p* < 0.0001, Figure 2b, Table S1). Moreover, when both PFLU*_3160* status and treatment were included in the same linear model, PFLU*_3160* status was a better predictor of fungicide MIC than treatment (LM Effect of *PFLU_3160* status F_1,20_ = 14.339, *p* = 0.001; NS effect of treatment F_2,22_ = 1.94, *p* > 0.05). Across all treatments, fungicide MIC of wildtype *PFLU_3160* clones ranged between 2 and 8 μg/mL, while clones harbouring a *PFLU_3160* mutation had increased MIC to 16–32 μg/mL (Table S1). Notably, the three fungicide‐evolved clones with MIC similar to ancestor (4–8 μg/mL) were also the only three fungicide‐evolved clones with wildtype *PFLU_3160* (Table S2). Together, this data supports the hypothesis that *PFLU_3160* mutations enhance fungicide resistance in our experimental populations.

### 3.4 Fungicide Selects for Increased Antimicrobial Resistance

**FIGURE 3:**
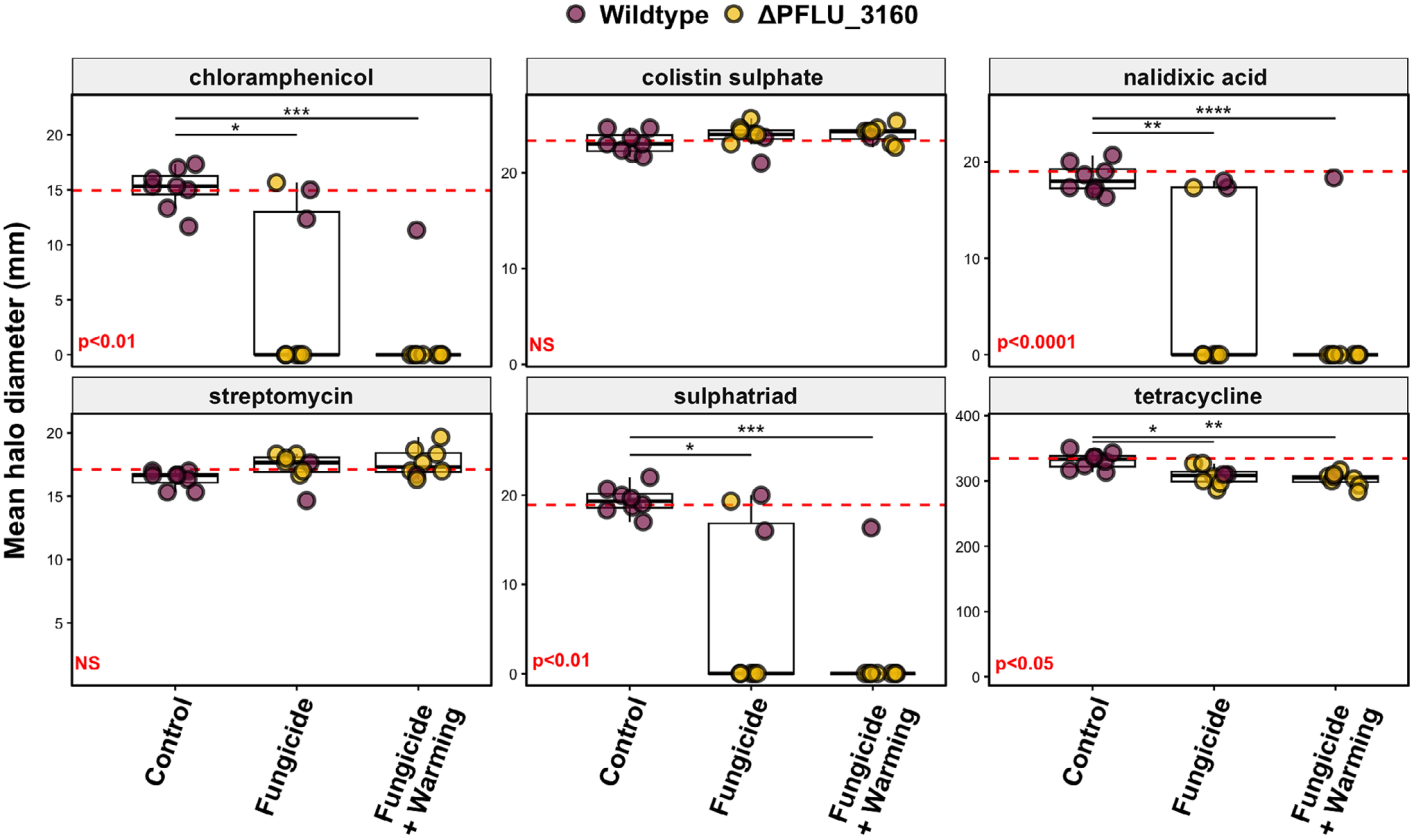
Mean halo diameter (mm) for *P. fluorescens* clones evolved under control, fungicide or fungicide+warming treatments, across six antimicrobials. Individual points represent mean halo diameter for 3 technical replicates per clone. Points are coloured according to whether that clone carries wildtype *PFLU_3160* or mutated *PFLU_3160*. Boxplots show the median (central line), interquartile range (box), and 1.5× interquartile range (whiskers). Dashed red horizontal line shows mean halo diameter (mm) for the ancestor. Lines connecting groups indicate significant differences based on Dunn posthoc tests. Overall Kruskal‐Wallis *p*‐values for each antibiotic are given in red. Significance levels are denoted as follows: *p* < 0.05 (*), *p* < 0.01 (**), *p* < 0.001 (***), *p* < 0.0001 (****). Red horizontal line shows mean value for 8 ancestral clones.

*PFLU_3160* is a *mexS* ortholog, and loss‐of‐function mutations in *mexS* have been implicated in conferring AMR in *Pseudomonas aeruginosa* (Fetar et al. 2011; Sobel et al. 2005). To test whether fungicide‐selected mutations induce cross‐resistance to antimicrobials, we performed antibiotic disk diffusion assays on the same sequenced clones subjected to fungicide resistance assays (section 3.3). Treatment‐specific differences in resistance were observed for chloramphenicol (Kruskal‐Wallis: χ^2^_2_ = 14.3, *p* < 0.01), sulphatriad (Kruskal‐Wallis: χ^2^_2_ = 13.7, *p* < 0.01), nalidixic acid (Kruskal‐Wallis: χ^2^_2_ = 24.7, *p* < 0.0001) and tetracycline (Kruskal‐Wallis: χ^2^_2_ = 12.5, *p* < 0.05) (Figure 3, Table S2). Across these 4 antibiotics, fungicide treatments (applied alone or in combination with warming) had increased resistance compared to controls (Dunn test; fungicide versus control: *p* < 0.05, fungicide+warming versus control: *p* < 0.05, for all four antibiotics). There was no difference in resistance between fungicide treatments evolved under warming versus control temperatures (Dunn test; fungicide versus fungicide+warming, *p* > 0.05 in all cases, Figure 3, Table S2). Hence, while fungicide increased resistance to 4 out of 6 tested antibiotics, this was unaffected by experimental warming. No treatment‐specific differences in resistance were observed for colistin sulphate (Kruskal‐Wallis: χ^2^_2_ = 2.3, *p* > 0.05) or streptomycin (Kruskal‐Wallis: χ^2^_2_ = 6.34, *p* > 0.05) (Figure 3, Table S2).

**FIGURE 4:**
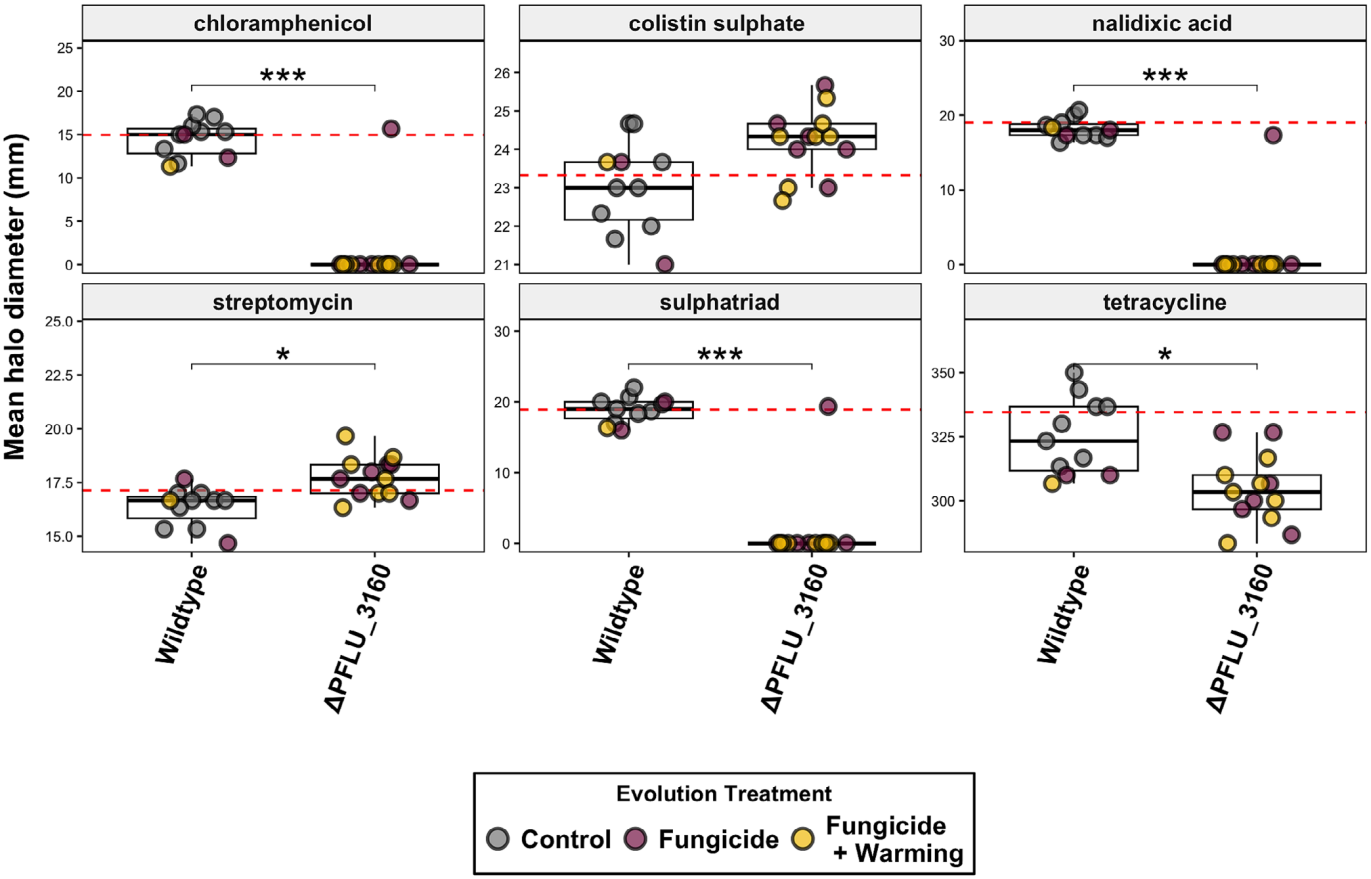
Antimicrobial susceptibility data from Figure 3, grouped by *PFLU_3160* mutation status (wildtype v mutated). For chloramphenicol, sulphatriad, nalidixic acid and tetracycline (Wilcox test; W = 124, *p* < 0.05) *PFLU_3160* mutants had significantly increased resistance versus wildtype. 11/12 Clones with mutations in *PFLU_3160* displayed complete resistance to chloramphenicol, nalidixic acid, and sulphatriad (i.e., zone of inhibition = 0), while all wildtype clones remained susceptible to these antibiotics. Conversely, for streptomycin, *PFLU_3160* mutants are more susceptible than wildtype clones, however this difference is very small (range of 1‐2 mm). Together, our data show fungicides can select for AMR, likely via selection for mutations in *PFLU_3160*.

We next directly compared antimicrobial susceptibility of clones harbouring a *PFLU_3160* mutation versus control evolved clones with wildtype *PFLU_3160*. For chloramphenicol (Wilcox test; W = 135, *p* < 0.001), sulphatriad (Wilcox test; W = 137, *p* < 0.001), nalidixic acid (Wilcox test; W = 555, *p* < 0.0001) and tetracycline (Wilcox test; W = 124, *p* < 0.05) *PFLU_3160* mutants had significantly increased resistance versus wildtype (Figure 4, Table S2). Eleven out of 12 *clones* with mutations in *PFLU_3160* displayed complete resistance to chloramphenicol, nalidixic acid, and sulphatriad (i.e., zone of inhibition =0), while all wildtype clones remained susceptible to these antibiotics. The one exception to this finding was clone B3X (the only clone with a mutation in *PFLU_3161*, a *mexT* ortholog), which harboured a mutation in *PFLU_3160*, yet remained susceptible to chloramphenicol, nalidixic acid and sulphatriad (Table S2). Conversely, for streptomycin (Wilcox test; W = 19.5, *p* < 0.05), *PFLU_3160* mutants were more susceptible than wildtype clones, however this difference was very small (range of 1‐2 mm) (Figure 4), so may not be clinically relevant. Together, our data show fungicides can select for AMR across multiple antibiotics (i.e., multidrug resistance), likely via selection for mutations in *PFLU_3160*.

## 4 Discussion

Using *P. fluorescens* experimental evolution in compost soil microcosms, we show that the fungicide Fubol Gold drives fungicide resistance and cross‐resistance to antibiotics chloramphenicol, nalidixic acid and sulphatriad, possibly by upregulating efflux activity and reducing outer membrane porin expression. Our observations align with a growing body of evidence showing that fungicides co‐select for AMR, even in the absence of antibiotic exposure (Murray et al. 2024). Experimental evolution in liquid media using *Escherichia coli* , shows that bacteria can adapt to a variety of fungicide classes through *de novo* mutations in genes involved in target site modification, membrane permeability and efflux activity, conferring cross resistance to antibiotics such as streptomycin, chloramphenicol and tetracycline (Xing et al. 2020; Yu et al. 2022). Our study differs from previous studies because we evolved bacteria in soil, rather than simple lab media. Nonetheless, our findings were strikingly similar: *P. fluorescens* became more resistant to fungicide after 16 weeks, and fungicide‐resistant clones were also completely resistant to chloramphenicol, nalidixic acid and sulphatriad (i.e., multidrug resistant).

Experiments using well‐characterised single bacterial species are valuable for capturing *de novo* evolutionary responses to fungicides. At the level of whole soil communities, fungicide‐driven selection for AMR has also been observed, mainly inferred through metagenomics data. Exposing soil communities to fungicide active ingredients (carbendazim, azoxystrobin or chlorothalonil) increases the prevalence of ARGs involved in antibiotic target replacement, drug inactivation and efflux mechanisms (Zhang et al. 2020) as well as the proportion of AMR bacteria in soil mesocosms (Aleksova et al. 2019). Fungicides can also increase dissemination of mobile genetic elements carrying ARGs, which can spread between strains (Zhang et al. 2023) and species (Song et al. 2023) in communities. For example, the fungicide active ingredients chlorothalonil, azoxystrobin and carbendazim increased the conjugative transfer of the antibiotic‐resistance plasmid RP4 between *E. coli* strains (Zhang et al. 2023). Mancozeb, one of the active ingredients of Fubol Gold, can accelerate transfer of the multidrug‐resistant plasmid RP4 from *E. coli* to *Pseudomonas putida* (Song et al. 2023). In natural microbial communities, plasmid dynamics are therefore likely to influence both the rate and trajectory of fungicide and antibiotic resistance evolution, which we could not capture in our single‐species soil microcosms. Moreover, species interactions more generally could also affect fungicide adaptation and hence co‐selection for AMR, due to trade‐offs in adapting to abiotic and biotic components of the environment (Scheuerl et al. 2020). Experiments (Xing et al. 2020; Yu et al. 2022) examining *de novo* adaptation to fungicides within a single species (including ours), together with studies comparing ARG prevalence in fungicide‐treated and control soils, support the idea that fungicides can drive AMR in soil bacteria. However, the relative contribution of different mechanisms for adaptation (e.g., horizontal gene transfer, *de novo* mutation or species sorting) remains unresolved. Future studies tracking both evolutionary change and horizontal gene transfer dynamics within complex microbial communities (perhaps approaches outlined (Montbel and Hrcek 2026)) would help disentangle the processes underlying fungicide‐associated AMR selection in soil.

We found strong evidence for selection on the *mexS* ortholog *PFLU_3160*. *mexS* represses *mexT*, inhibiting the production of the *MexEF‐OprN* multidrug efflux system (Köhler et al. 1999; Sobel et al. 2005; Uwate et al. 2013) and increasing the expression of OprD porins (Ochs et al. 1999). Previous work in *P. aeruginosa* has also shown that overexpression of MexEF‐OprN efflux pumps together with reduced expression of OprD porins can increase antibiotic resistance through a combination of enhanced efflux and reduced membrane permeability (Chuanchuen et al. 2001; Fetar et al. 2011; Huang and Hancock 1993; Ochs et al. 1999; Sobel et al. 2005; Uwate et al. 2013). Based on the homology between *PFLU_3160* and *mexS*, our results suggest that similar mechanisms may contribute to the phenotypes observed here. However, *mexS* mutations have not previously been linked to fungicide resistance. One of our evolved clones, B3X, was the only PFLU_3160 mutant with increased fungicide resistance without a corresponding increase in antibiotic resistance. B3X was also the only clone with a mutation (SNP) in *PFLU_3161*, a *mexT* ortholog. This SNP may have altered *MexT* structure, preventing the increased expression of MexEF‐OprN while still reducing OprD porin expression, putatively explaining the observed phenotype of increased fungicide resistance (via reduction expression of OprD porins) but not antibiotic resistance. Our genomic results align with similar studies on *E.coli*, where the fungicide copper hydroxide caused reduced expression of outer membrane porins and increased expression of multidrug efflux pumps (Yu et al. 2022). However, we acknowledge that our finding is limited to mutational analyses rather than direct phenotypic tests on efflux activity, and we did not directly test the effect of the *mexS* mutation on resistance, e.g., using knockout mutants.

We did not see any clear effect of warming on the pattern of mutations, despite a warming regime that reached 34°C, which is beyond the optimal temperature range for environmental strains of *P. fluorescens*, such as SBW25 (Donnarumma et al. 2010). However, we note that short‐read whole genome sequencing does not capture all genetic variation, particularly structural variants, low‐frequency mutations or mutations in repetitive regions (Alkan et al. 2011). The absence of warming‐specific mutations does not show that warming had no effect on evolutionary trajectories, only that we could not detect clear genomic changes under our experimental conditions.

Mutations in *actP*, an acetate‐scavenging transporter (Gimenez et al. 2003) were widespread in fungicide‐free control populations but were never detected in fungicide‐free populations. We identified SNPs, insertions and large deletions in *actP* or *actP*‐adjacent regions, consistent with loss‐of‐function. A previous study using the same *P. fluorescens* strain and experimental compost system showed that *actP* mutations are beneficial in soil per se, yet are similarly constrained by the presence of a competing species, *P. putida* (Hall et al. 2018). Why *actP* remains intact in the presence of fungicide (this study) or a competitor (Hall et al. 2018), remains unclear. In *Neisseria meningitidis* , acetate can protect against oxidative stress (Haldar et al. 2025), suggesting that in *P. fluorescens* , acetate uptake via *actP* might help buffer cells against reactive oxygen species generated by mancozeb (a component of Fubol Gold) (Calviello et al. 2006) or by metabolic stress arising from nutrient competition (Calviello et al. 2006). One caveat is that we sequenced clones from the last viable timepoint of our evolution experiment, which was earlier for fungicide‐evolved populations. Since fungicide‐free clones had a longer period of evolution (and on average, higher population densities), it is possible that *actP* mutations would have eventually evolved in fungicide‐treated populations. However, given that Hall et al. (Hall et al. 2018) found similarly strong selection for *actP* mutations in the absence, but not presence of a (biotic) stressor, and the clear environment‐dependent benefits of *actP* mutations quantified by (Hall et al. 2018), there is good support for the general role of environmental stress‐dependent fitness effects of *actP*.

As climate change and other anthropogenic pressures generate persistent environmental disturbances, an important question is whether local populations can persist under progressively deteriorating conditions, such as those tested in our study. If population absolute fitness becomes negative under multiple stressors, populations are predicted to decline towards extinction (Bell 2017). However, evolutionary adaptation can allow populations to avoid extinction, if beneficial mutations increase absolute fitness from negative to positive, a process known as evolutionary rescue (Smith et al. 2022). In our study, clones from all fungicide‐treated populations evolved increased fungicide resistance, irrespective of warming. However, despite similar levels of fungicide resistance and patterns of mutation between warmed and unwarmed populations, evolutionary rescue was more effective when fungicide was applied as a single stressor, compared to when it was applied alongside warming (i.e., populations went extinct much earlier in the latter). One possible explanation is that the costs of fungicide resistance increased with temperature over the course of the experiment, as observed previously for bacteriophages (Quance and Travisano 2009) and antibiotics (Herren and Baym 2022). While this explanation is speculative, our results show that multistressor environments can have hidden effects on fitness and evolutionary rescue, even when evolutionary trajectories appear similar. Our findings highlight the importance of incorporating multiple, interacting environmental variables when predicting microbial responses to global change.

## Funding

This work was supported by the Natural Environment Research Council, NE/S00713X/1. Medical Research Council, MR/W02666X/1. Biotechnology and Biological Sciences Research Council, BB/T009446/1.

## Conflicts of Interest

The authors declare no conflicts of interest.

## Supporting information


**Supporting Information: S1** List of genomic mutations associated with evolved and ancestral *P. fluorescens* clones.

**Supporting Information: S2** Full experimental dataset.

**Figure S1:** The effect of four different fungicides on final densities of *P. fluorescens* in soil microcosms (Log_10_ CFU g^−1^). All fungicide formulations significantly decreased *P. fluorescens* densities compared to fungicide‐free controls (see main text for statistics). Assays were replicated in triplicate. Asterisks denote significant pairwise t‐test comparisons, with Bonferroni adjustment.
**Figure S2:** (A) Kaplan–Meier survival curves showing the survival probability of experimental populations over time under four treatments: control (no warming, no fungicide), fungicide only, warming only, and dual stress (warming + fungicide). Dual‐stress populations went extinct earliest and most completely, whereas single‐stressor populations experienced intermediate losses, and all control populations remained viable. See main text for statistics. (B) Fungicide concentrations in soil increased every 4 weeks, to a maximum of 500 mg kg−1 at weeks 12–16. (C) In warming treatments, temperatures increased by 0.5°C weekly until a maximum temperature of 34°C by week 16.
**Figure S3:** Population densities (Log10 CFU g−1 soil) over the course of a 16‐week evolution experiment. Populations were evolved under control conditions (A), with fungicide (B), warming (C) or both fungicide and warming (D). Individual lines show densities for a single evolving population in soil. Fungicide concentrations in soil increased every 4 weeks, to a maximum of 500 mg kg−1 at weeks 12–16 (E). In warming treatments, temperatures increased by 0.5°C weekly until a maximum temperature of 34°C by week 16 (F). Dual‐stressor populations showed the greatest decline in population densities, with extinctions as early as week 8.
**Figure S4:** To identify treatment‐specific patterns of mutation, we sequenced the genome of two clones from each evolved population (Clone ID; y‐axis). Locus tag/gene name is shown on x‐axis. Clones are separated by treatment in which they evolved (Fungicide, Warming, Fungicide+Warming, Control). Across evolved clones, we detected 152 mutations comprising gene deletions (triangles), insertions (circles) and single nucleotide polymorphisms (squares). Evolved clones had between 0 and 6 mutations with a median of 2 mutations per clone. We did not detect any mutations in one clone (B8Y; fungicide‐only treatment). Two targets consisted of large deletions in repeat‐rich intergenic regions, which are prone to spurious mutation calls and should be interpreted with caution (PFLU_2441‐PFLU_2442 and PFLU_4254‐ PFLU_4255). Colour corresponds to a particular replicate population. Further information on specific mutations is available in S1.
**Table S1:** Mode fungicide minimum inhibitory concentrations (MIC) for evolved and all 8 ancestral P. fluorescens clones. MIC's are denoted via an orb colour, with green being the most resistant and red being the least resistant to fungicide, respectively. All clones were subjected to whole genome resequencing and details on PFLU_3160 (mexS ortholog) mutation status is given. All clones harbouring a PFLU_3160 mutation have increased resistance to fungicide. Mutation type: DEL = Deletion; SNP = Single Nucleotide Polymorphism; INS = Insertion; − = no mutation.
**Table S2:** Antimicrobial zone of inhibition in mm (ZOI) for evolved and ancestral P. fluorescens clones. Resistance level is denoted via an orb colour, with green being the most susceptible and red being completely resistant, respectively. Harbouring mutations in PFLU_3160 are associated with complete resistance (ZOI = 0 mm) to Chloramphenicol, Sulphatriad and Nalidixic acid. The following disc concentrations were used: Chloramphenicol (30 μg), Sulphatriad (200 μg), Tetracycline (25 μg), Nalidixic acid (30 μg). Mutation type: DEL = Deletion; SNP = Single Nucleotide Polymorphism; INS = Insertion; − = no mutation. All ancestral clones were within the same range as control clones.

**Data S1:** Supporting Information.

## Data Availability

Data for this study are available in the Supporting Information.
